# Medical Intelligence Department

**Published:** 1918-08

**Authors:** 


### MEDICAL INTELLIGENCE DEPARTMENT

In furtherance of his plans for increasing the medical opportunities of American physicians in France, Major Alexander Lambert, Medical Adviser of the A. R. C. in France, has established two bureaus. One of these — that in his office at 12 Place Vendôme — aims to give to visiting medical men full information as to the medical and surgical work in Paris, not alone in direct connection with the army and its medico-surgical problems, but especially the research work in every department that is being done by French physicians. This bureau has been put in charge of Dr. D. T. Boulanger who has for some years been doing research work in America in connexion with X-ray and radium. Dr. Boulanger will not only be able to give to physicians full data about all clinics, but will endeavor to facilitate the opportunity of making full use of the clinics by personally conducting those unfamiliar with Paris, so that a minimum of time may be lost by men whose visits are short.

The other bureau, under the charge of Dr. Thomas H. Halsted, at 9 Rue du Mont-Thabor, is designed to bring the medical and surgical personnel of the A. E. F. into closer relation to the advances in medicine, so that any medical officer in the army anywhere in France can write or telegraph to this office for the last word on any subject and have it sent out at once.

In the meantime a force of workers is abstracting and filing articles on war subjects for library and circulating purposes so as to conserve the time of medical men in keeping abreast of all important advances.

